# Genetics of smoking and risk of clonal hematopoiesis

**DOI:** 10.1038/s41598-022-09604-z

**Published:** 2022-05-04

**Authors:** Michael G. Levin, Tetsushi Nakao, Seyedeh M. Zekavat, Satoshi Koyama, Alexander G. Bick, Abhishek Niroula, Benjamin Ebert, Scott M. Damrauer, Pradeep Natarajan

**Affiliations:** 1 Division of Cardiovascular Medicine, Department of Medicine, University of Pennsylvania Perelman School of Medicine, Philadelphia, PA USA; 2 Corporal Michael J. Crescenz VA Medical Center, Philadelphia, PA USA; 3 Program in Medical and Population Genetics and Cardiovascular Disease Initiative, Broad Institute of Harvard and MIT, Cambridge, MA USA; 4 Cardiovascular Research Center, Massachusetts General Hospital, 185 Cambridge Street, CPZN 3.184, Boston, MA 02114 USA; 5 Department of Medical Oncology, Dana-Farber Cancer Institute, Boston, MA USA; 6 Division of Cardiovascular Medicine, Department of Medicine, Brigham and Women’s Hospital, Boston, MA USA; 7 Yale University School of Medicine, New Haven, CT USA; 8 Division of Genetic Medicine, Department of Medicine, Vanderbilt University School of Medicine, Nashville, TN USA; 9 Cancer Program, Broad Institute of Harvard and MIT, Cambridge, MA USA; 10 Department of Laboratory Medicine, Lund University, Lund, Sweden; 11 Howard Hughes Medical Institute, Boston, MA USA; 12 Department of Surgery, University of Pennsylvania Perelman School of Medicine, Philadelphia, PA USA; 13 Department of Medicine, Harvard Medical School, Boston, MA USA

**Keywords:** Risk factors, Haematological cancer, Cancer genomics, Population genetics

## Abstract

Clonal hematopoiesis of indeterminate potential (CHIP) and mosaic chromosomal alterations (mCAs) represent two forms of clonal hematopoiesis where clones bearing expanded somatic mutations have been linked to both oncologic and non-oncologic clinical outcomes including atherosclerosis and all-cause mortality. Epidemiologic studies have highlighted smoking as an important driver of somatic mutations across multiple tissues. However, establishing the causal role of smoking in clonal hematopoiesis has been limited by observational study designs, which may suffer from confounding and reverse-causality. We performed two complementary analyses to investigate the role of smoking in mCAs and CHIP. First, using an observational study design among UK Biobank participants, we confirmed strong associations between smoking and mCAs. Second, using two-sample Mendelian randomization, smoking was strongly associated with mCA but not with CHIP. Overall, these results support a causal association between smoking and mCAs and suggest smoking may variably shape the fitness of clones bearing somatic mutations.

Population-based human genetic analyses of asymptomatic individuals have shown that clonally-expanded acquired mutations in the blood are increasingly common with age^1–5^. Clonal hematopoiesis of indeterminate potential (CHIP) is characterized by hematologic expansion of clones bearing pathogenic single nucleotide polymorphisms or small insertions/deletions, typically in *DNMT3A*, *TET2*, *ASXL1*, *JAK2*, and other oncogenic genes, detected by next-generation sequencing^6^. Mosaic chromosomal alterations (mCAs) are characterized by expanded large structural variants, 50–249 Mb, often detected by genome-wide array genotyping^3^. CHIP is strongly linked to myeloid malignancy and atherosclerotic cardiovascular disease^2,7–9^, while mCAs are strongly linked to lymphoid malignancies, myeloproliferative neoplasms, and severe infections^10–12^.

While tobacco smoking is an established strong mutagen^13^, observational associations between smoking and somatic mutations may be limited by residual confounding or reverse-causality, precluding causal inference. Smoking has been linked to mosaic loss-of-Y chromosome (LOY)^14^, as well as loss-of-function mutations in *ASXL1*, a CHIP-associated tumor-suppressor gene^15^. However, the broader relationships between smoking and variants indicative of clonal hematopoiesis, and the extent to which residual confounding from correlated lifestyle factors may explain the observed relationships is not well understood.

Genome-wide association studies (GWAS) have recently identified common germline genetic variants associated with smoking, mCA, and CHIP, enabling causal inference across these traits within the Mendelian randomization (MR) framework^12,16–18^. Because genetic variants are randomly allocated at conception, these variants may be used as instrumental variables for MR, which under certain assumptions enables a natural experiment mimicking a randomized controlled trial^19^. Compared with traditional observational study designs, MR studies may be less susceptible to confounding and reverse causality, enabling estimation of putative causal associations between exposures and outcomes^19^.

We aimed to (1) confirm observational associations between smoking and common manifestations of somatic mutation (mCA and CHIP), and (2) estimate putative causal associations between smoking and these outcomes within the MR framework.

In our observational analysis, mCAs were genotyped in 487,279 participants of UK Biobank who underwent genome-wide genotyping of blood DNA, and CHIP was genotyped in 49,606 participants who underwent whole-exome sequencing of blood DNA^3,8,17^. We identified 72,176 (14.8%) individuals with any mCA, 17,108 (3.5%) with autosomal mCA, and 44,696 (20% of males) with mCA-LOY. Among the 49,606 individuals who underwent whole exome sequencing, 2888 (5.8%) had CHIP. Smoking was independently associated with any mCA (OR 1.24, 95% CI 1.21–1.26, *p* = 8.55 × 10^–131^), as well as subclasses: autosomal mCA (OR 1.08, 95% CI 1.05–1.12, *p* = 6 × 10^–7^), mCA-LOX (OR 1.05, 95% CI 1.01–1.08, *p* = 0.02), mCA-LOY (OR 1.35, 95% CI 1.32–1.39, *p* = 8.16 × 10^–154^), Myeloid mCA (OR 1.16, 95% CI 1.07–1.26, *p* = 2 × 10^–4^), and Lymphoid mCA (OR 1.09, 95% CI 1.02–1.16, *p* = 0.008) (Fig. 1). There was substantial heterogeneity in the associations between smoking and mCA subclasses (I^2^ = 97.7, Cochran Q = 218.5, *p* = 3.06 × 10^–45^), with the association between smoking and overall mCA likely driven by mCA-LOY. Smoking was also associated with CHIP overall (OR 1.15, 95% CI 1.06–1.24, *p* = 4.64 × 10^–4^) (Fig. 1). In secondary analyses we tested for associations between smoking and CHIP by gene (e.g., *DNMT3A*, *TET2*, *ASXL1*, etc.). However, there was significant heterogeneity when considering associations between smoking and mutations in specific CHIP-associated genes (I^2^ = 54.4, Cochran Q = 19.7, *p* = 0.02). These observational results were similar in sensitivity analyses accounting for alcohol consumption. Broadly, these findings are consistent with prior reports correlating smoking status with increased risk of somatic mutations^4,13,14,20,21^.

**Figure 1:**
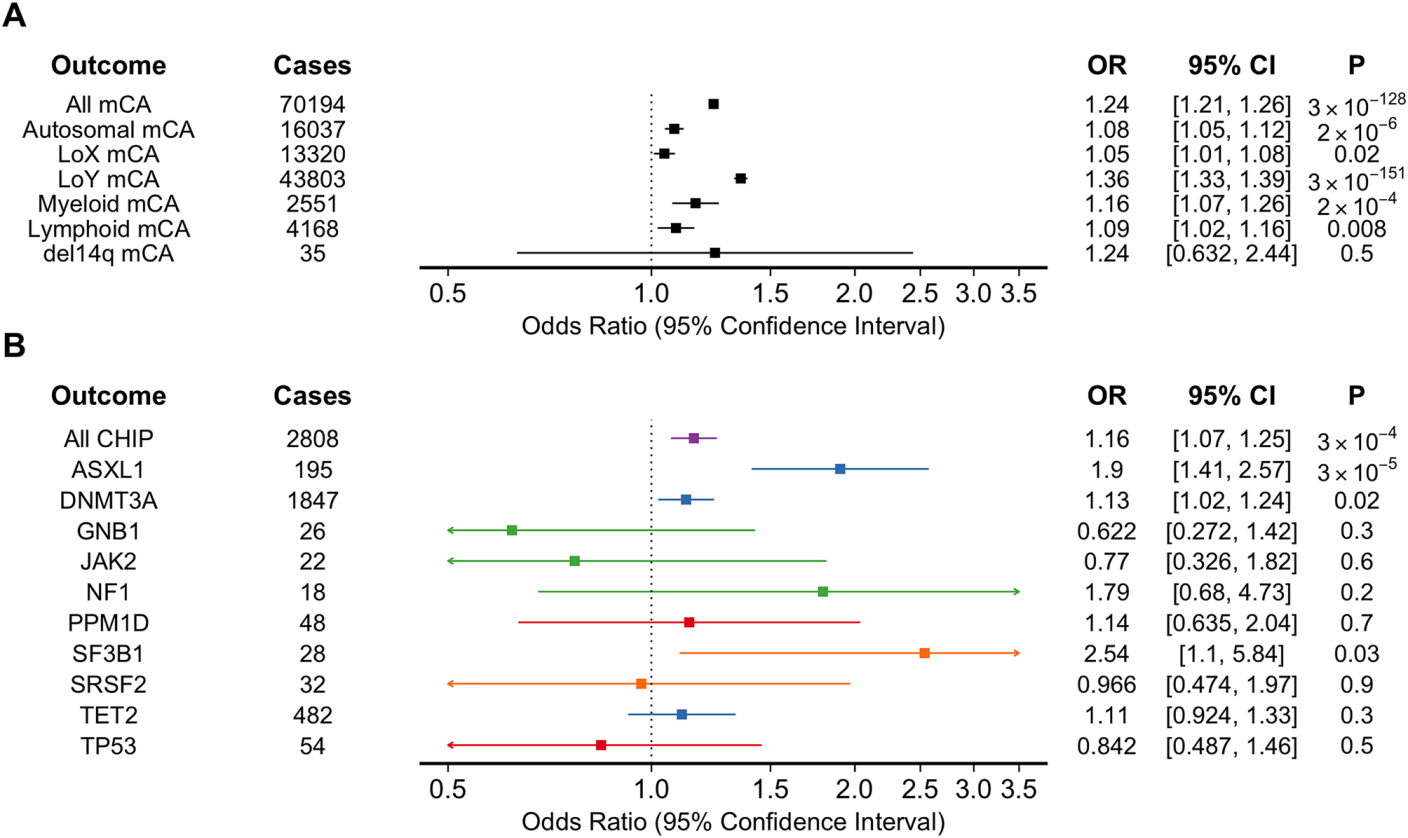
Observational associations between Ever smoking and somatic mutations among UK Biobank participants. Associations between ever smoking and (**A**) manifestations of mCA and (**B**) manifestations of CHIP among UK Biobank participants. Colors represent specific types of CHIP mutations (Purple = any CHIP, Blue = epigenetic, Red = DNA damage repair, Orange = splicing, Green = other). mCA = mosaic chromosomal alteration; LoX = loss-of-X chromosome; LoY = loss-of-Y chromosome; del = deletion.

Next, we performed two-sample MR using summary statistics to evaluate the causal effects of smoking on somatic mutation outcomes. As a genetic proxy for smoking, we considered up to 119 independent genetic variants associated with smoking at genome-wide significance (p < 5 × 10^–8^)^16^. The F-statistic for our genetic instrument ranged from 21.78 to 196 (mean 48.5), suggesting the analysis was not limited by weak-instrument bias. In the primary inverse variance-weighted MR analysis, smoking was strongly associated with mCAs (OR 1.44, 95% CI 1.16–1.79, *p* = 8 × 10^–4^), and mCA-LOY (OR 1.06, 95% CI 1.04–1.08, *p* = 1 × 10^–8^) (Fig. 2). We did not detect a significant association between smoking and CHIP (Transethnic OR 1.01, 95% CI 0.74–1.37, *p* = 1; European [EUR] OR 0.70, 95% CI 0.48–1.02, *p* = 0.06), however these confidence intervals do not exclude potentially meaningful effects (Fig. 2). Results were similar using alternative MR methods which each make different assumptions about outliers and pleiotropy (Fig. 2). The MR-Egger bias intercept test did not detect evidence of directional pleiotropy (*p* > 0.05 for all comparisons).

**Figure 2:**
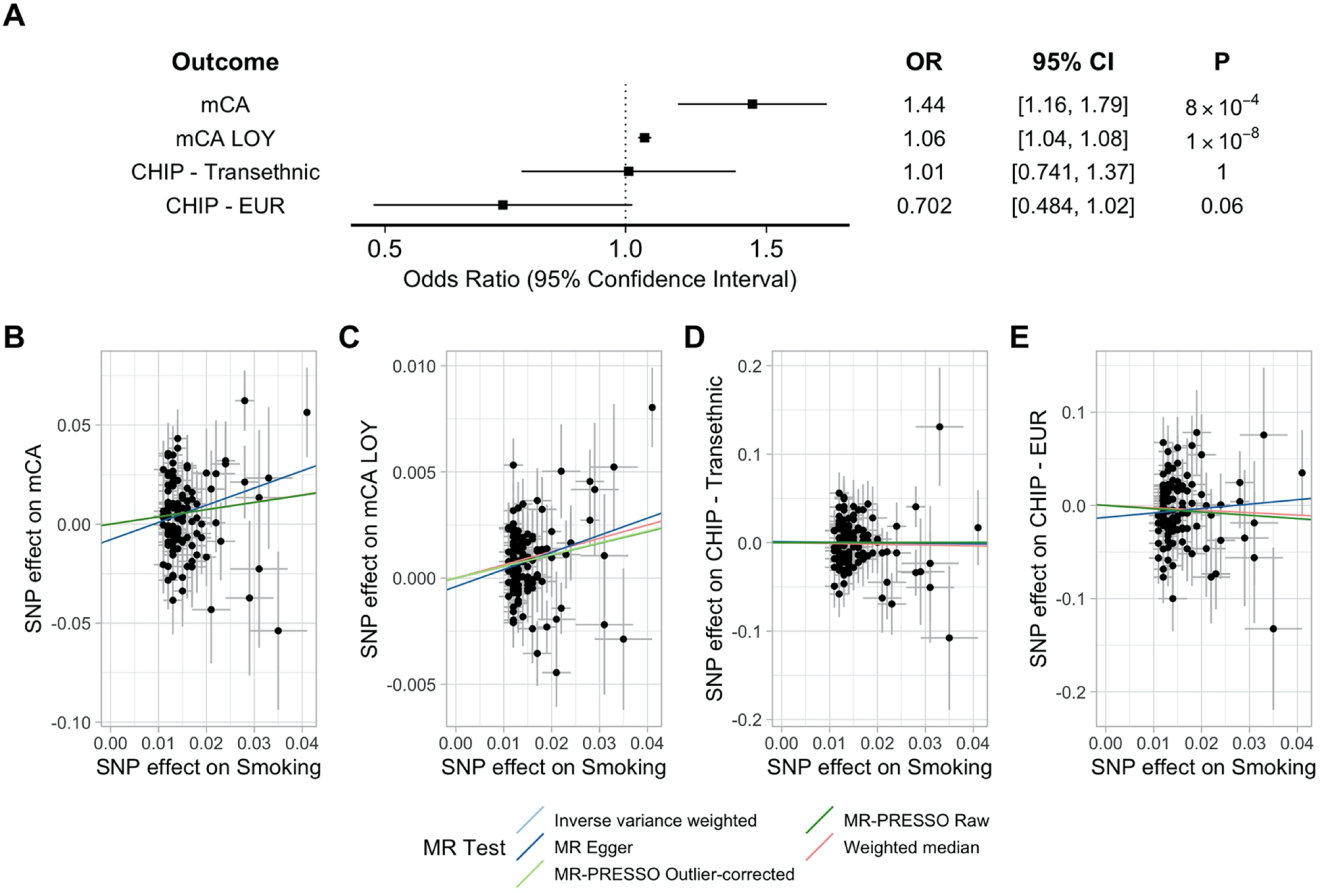
Mendelian Randomization associations between smoking and somatic mutations. Results of MR testing the associations between smoking and mCA and CHIP outcomes. (**A**) Inverse-variance weighted MR results. (**B**–**E**) MR results testing the association between smoking and each outcome using alternative MR methods. EUR = European-ancestry.

Overall, these results are consistent with smoking as a causal risk factor for mCA. Although smoking was associated with CHIP in our observational analysis, we did not detect an association in our MR analysis; whether smoking represents a causal risk factor for CHIP, including for specific genes, will require further study. Our findings suggest that smoking variably shapes the fitness of distinct somatic mutations, and efforts to reduce smoking would be expected to reduce the burden of the downstream consequences of somatic mutation.

This study has both strengths and limitations. In this case, the MR framework allowed us to leverage the natural randomization in the distribution of genetic variants to estimate the causal associations between smoking and manifestations of somatic mutations. By utilizing large GWAS, we were able to consider thousands of mCA and CHIP cases, which would otherwise require large, extended trials to accrue. Although we were able to identify strong associations between smoking and mCA, whether mCA mediates some of the adverse consequences of smoking will require further study. Similarly, we do not provide specific insights regarding the mechanisms by which smoking influences fitness of clones bearing somatic mutations. Given the low heritability of CHIP, and small sample size of CHIP cases limiting power, we cannot exclude meaningful associations between smoking and CHIP by gene in an MR framework. Smoking has been linked to the fitness of clones harboring mutations in particular driver mutations in the setting of lung cancer^13^, and whether similar findings extend to CHIP remains an important avenue for future study. With the growing availability of population-scale human genotype and sequence data, linking smoking to particular mutational drivers of clonal fitness should become increasingly tractable.

In conclusion, we confirm strong observational associations between smoking and somatic mutation, with MR analyses consistent with smoking as a causal risk factor for mCA. Whether smoking causes CHIP, and the specific mechanisms by which smoking influences fitness will require further study.

## Methods

### Observational analysis

Whole exome sequencing and array genotyping have been previously described in the UK Biobank, a population-based volunteer biobank recruited 2006–2010^22^. mCA was determined among 479,810 participants without hematologic malignancy who underwent genome-wide genotyping, and CHIP was determined among up to 48,966 participants without hematologic malignancy who underwent whole-exome sequencing, as previously described^3,8,17^. We tested for the association between ever smoking (defined by UK Biobank unique data identifier 20116-0.0) and somatic mutation outcomes (mCA, autosomal mCA, mCA-LOY [loss-of-Y chromosome], mCA-LOX [loss-of-X chromosome], lymphoid mCA, myeloid mCA, del14q, and CHIP) using logistic regression adjusted for age, age^2^, sex, sequencing batch, and 15 genetic principal components. Myeloid and lymphoid mCAs were identified based on their association with myeloid and lymphoid malignancies^23^.﻿ In a secondary analysis we tested for associations between smoking and CHIP by gene (e.g., *DNMT3A*, *TET2*, *ASXL1*, etc.). In a sensitivity analysis we included self-reported alcohol use as an additional covariate, given strong epidemiologic correlations with smoking. This work was performed using UK Biobank Application #7089. The UK Biobank obtained IRB approval from the North West Multi-centre Research Ethics Committee (approval number: 11/NW/0382), and participants provided informed consent.

### Mendelian randomization analysis

We performed two-sample MR using summary statistics utilizing the *TwoSampleMR* package in R. As genetic instruments for smoking, we considered independent (r^2^ < 0.001, distance > 10,000 kb) genetic variants associated (*p* < 5 × 10^–8^) with the lifetime smoking index, a previously-validated continuous measure of lifetime smoking, derived among up to 462,690 UK Biobank participants^16^. For each genetic variant associated with smoking, we extracted the corresponding effects for each outcome from GWAS of mCA (up to 767,891 unrelated multi-ancestry individuals without hematological cancer from UK Biobank, Biobank Japan, Mass General Brigham Biobank, and FinnGen), mCA-LOY (up to 205,011 male participants of UK Biobank), and CHIP (97,691 participants of TOPMed)^12,17,18^. We calculated F-statistics for each exposure-outcome pair to assess for weak-instrument bias^19^. For the primary analysis we applied the inverse variance-weighted method, but considered weighted median, MR-Egger, and MR-PRESSO in sensitivity analyses, as these methods make different assumptions about the presence of pleiotropy and outliers^24^.

Analyses were performed using R 4.0.3 (R Foundation for Statistical Computing, Vienna, Austria). For the primary observational and MR analyses, *p* values < 0.05 (after accounting for multiple comparisons using Bonferroni adjustment) were considered significant. For secondary analyses, *p* < 0.05 was considered significant. All methods were carried out in accordance with the relevant guidelines and regulations.
